# Diffusible Signal Factors and *Xylella fastidiosa*: A Crucial Mechanism Yet to Be Revealed

**DOI:** 10.3390/biology14030303

**Published:** 2025-03-17

**Authors:** Letizia Portaccio, Marzia Vergine, Mariarosaria De Pascali, Luigi De Bellis, Andrea Luvisi

**Affiliations:** 1 Department of Biological and Environmental Sciences and Technologies, University of Salento, 73100 Lecce, Italy; letizia.portaccio@unisalento.it (L.P.); mariarosaria.depascali@unisalento.it (M.D.P.); luigi.debellis@unisalento.it (L.D.B.); andrea.luvisi@unisalento.it (A.L.); 2 National Biodiversity Future Center, 90133 Palermo, Italy

**Keywords:** quorum sensing, control strategies, biofilm, virulence, signal molecules and bacteria

## Abstract

*Xylella fastidiosa* (*Xf*) is a xylem-limited Gram-negative phytopathogen responsible for severe plant diseases globally. Colonization and dissemination on host plants are regulated primarily by diffusible signal factors (DSFs) and quorum sensing (QS) molecules regulating biofilm formation, motility, and virulence factor synthesis. DSFs play a critical role in the transition of bacteria from adhesion to dispersal phases, influencing plant infection and transmission by vector. Because of *Xf*’s host range (over 550 plant species), effective containment strategies are highly demanded. In this review, we discuss the molecular mechanism of DSF-mediated signalling in *Xf*, especially concerning its role in pathogenicity and adaptation. Moreover, we shed light on innovative approaches to manage *Xf*, including quorum-quenching (QQ) strategies and transgenic plants targeted to disrupt QS pathways. Improved knowledge of DSF interactions with host plants and bacterial communities could provide an entry point for novel, sustainable disease control strategies to decrease *Xf*’s agricultural and ecological impact.

## Simple Summary

*Xylella fastidiosa* is a plant pathogenic bacterium that employs diffusible signal factors as part of its quorum sensing system to regulate biofilm formation, motility, and the expression of virulence factors. Unlike other bacterial communication systems, these signals play a dual role: they enhance adhesion within the xylem vessels of host plants while facilitating dispersal through insect vectors. This unique mechanism is crucial for the bacterium’s spread and persistence. Innovative control strategies include quorum quenching, which disrupts the signaling process to suppress bacterial virulence, as well as the development of genetically modified plants that produce high levels of diffusible signal factors to alter bacterial behaviour. These approaches provide more sustainable and environmentally friendly alternatives to conventional control methods. However, further research is essential to gain a better understanding of the molecular interactions between diffusible signal factors and host plants, identify new enzymes capable of breaking down these signals, and refine biotechnological strategies to interfere with bacterial communication. Advancing our knowledge in this area could lead to long-term solutions for reducing infections and enhancing crop resistance against this destructive pathogen.

## 1. Introduction

**Figure 1:**
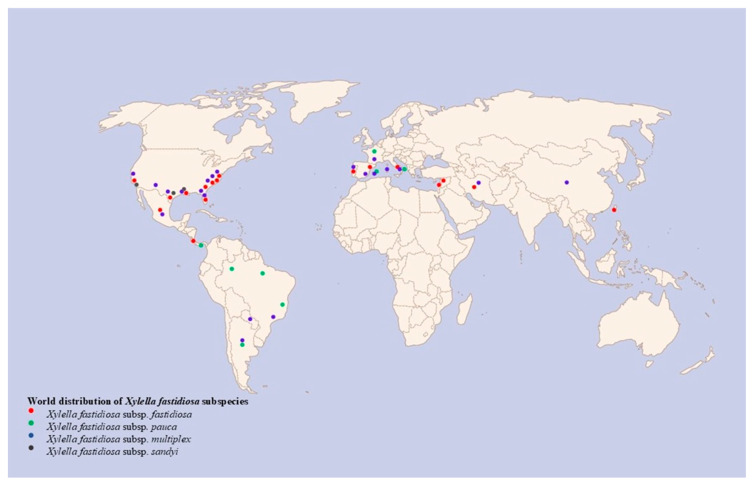
Global Distribution of *Xylella fastidiosa* subspecies.

*Xylella fastidiosa* (*Xf*) is a Gram-negative Xanthomonadaceae bacterium with rod-shaped morphology. The disease is transmitted primarily by sap-feeding insects like spittlebugs and leafhoppers, where the bacterium is moved from infected to healthy plants [1,2]. Once inside the xylem, *Xf* exploits the nutrient-poor environment by forming biofilms that block water and nutrient transport, leading to symptoms such as leaf scorch, stunted growth, and plant death [1]. The survival and development of the bacterium under these conditions are accounted for by its unique adaptations, including the synthesis of diffusible signal factors (DSFs) that play a crucial role in its quorum sensing (QS) mechanisms [1,3]. Its diversity in host plants is considerable, with more than 550 hosts and some of the subspecies, such as *pauca*, *fastidiosa*, *multiplex*, and *sandyi*, (Figure 1), being associated with specific plant diseases [2,4].

For instance, the disease caused by *Xf* subsp. *pauca*, detected for the first time in Apulia in 2013 on olive trees [5], progresses at varying rates depending on local conditions, as evidenced by regional data from Apulia. Studies show that it advances at approximately 20 km per year [6], while another analysis based on logistic models estimated a slower spread rate of about 10 km per year [7].

Research from the European Food Safety Authority (EFSA) has further highlighted the alarming spread of this pathogen. The data indicates that 90% of newly infected trees within a year are located within 5.2 km of previously infected areas [7].

Recent scientific studies have provided updated insights into the spread of *Xf* in the Apulia region of Italy. A 2024 study analysing data from 2020 to 2023 found that the incidence of *Xf* in containment and buffer zones remained low, ranging from 0.06% to 0.70% among sampled plants [8].

Regarding cultivar susceptibility, some olive varieties are more vulnerable to the disease than others. The Ogliarola salentina and Cellina di Nardò cultivars are highly susceptible. In contrast, the Leccino and FS-17^®^ cultivars demonstrate a higher degree of resistance, with significantly fewer symptoms and lower bacterial density in infected tissues [9]. The resistance of Leccino, in particular, is thought to be associated with increased lignin content in its xylem vessels, which hinders pathogen movement [10]. Additionally, the presence of phenolic compounds [11] and narrower xylem vessels in these cultivars helps to reduce the risk of embolism, which could otherwise exacerbate disease spread [12].

However, cultivar susceptibility does not solely influence the spread of *Xf.* The abundance of cultivated and wild host species in the affected area is a critical factor in its ongoing spread. Many of these plants serve as alternative hosts or reservoirs for the pathogen [13]. Coupled with the activity of insect vectors such as *Philaenus spumarius*, which can spread the pathogen between plants, these factors play a crucial role in the disease’s persistence and expansion [13,14].

*Xf* is considered a quarantine pathogen, and there are no effective chemical control methods [15]; therefore, the mode of action of signal molecules produced by *Xf* can be a significant area of investigation to evaluate the possibility of their exploitation to develop targeted containment strategies. DSFs, cis-2 unsaturated signal molecules with a double bond [16,17], are the target of *Xf*’s QS system. This regulates important bacterial processes, including the production of virulence factors, biofilm formation, and antibiotic resistance [18,19].

Fatty acids like DSFs are significant constituents of cellular architecture and play a role in phospholipid biosynthesis, membrane permeability, and bacterial virulence [20,21]. They are synthesized in plastids through the action of enzymes such as acetyl-CoA carboxylase and fatty acid synthase [22]. Understanding DSF interactions with host defence mechanisms and how such molecules influence development hold great promise strategies to reduce the spread of *Xf* [1,3].

Therefore, this review sets out to outline recent research within this area, shed new light on the role of DSFs and whether they could act as a substrate for novel containment strategies.

## 2. Diffusible Signal Factors (DSFs)

### 2.1. Quorum Sensing and DSF Molecules: Mechanisms and Their Role in Bacterial Communication

QS is a complex communication system used by bacteria to coordinate their behaviour according to population density. QS is required to allow bacterial cells to respond to their environment through the coordinated regulation of gene expression. Bacteria secrete small amounts of signalling molecules referred to as autoinducers at low cell densities. They include acyl-homoserine lactones (AHLs) for Gram-negative bacteria [3,23,24] and other molecules referred to as autoinducers-2 products of 4,5-dihydroxy-2,3-pentonedione [25,26]. As the population increases, the concentration of these signals rises, leading to significant changes in gene expression that regulate various functions, such as biofilm formation, virulence factor production, and interspecies competition [27]. The mechanism of AHL-mediated gene regulation involves a transcriptional regulator (R protein) and an AHL synthase (I protein), where the binding of AHL to the R protein activates specific gene expression [1,28].

The first DSF was discovered in *Xanthomonas campestris* pv. *campestris* (*Xcc*) and is recognised as cis-11-methyl-dodecenoic acid. This bacterium also produces several other DSFs, including cis-2-dodecenoic acid and cis-10-methyl-2-dodecenoic acid, all of which are unsaturated fatty acids characterised by a carbon chain and a cis-double bond [29,30,31].

The cis-double bond is critical for recognising and activating the receptor RpfC, ensuring precise communication within bacterial populations and minimising non-specific interactions [32]. The discovery of DSFs was driven by molecular genetic analyses aimed at understanding the regulation of extracellular degrading enzymes and extracellular polysaccharide (EPS) virulence factors in *X. campestris* [33,34]. Anyway, DSFs are not exclusive to *Xanthomonas* species; they are also produced by other Gram-negative plant pathogens, including *Xanthomonas oryzae* and *Burkholderia* species [35,36]. Additionally, *Xf* also produces DSFs that play a role in its pathogenicity [37]. The signalling pathways involving DSFs are essential for the expression of virulence factors and the formation of biofilms, which are critical for the survival and spread of these pathogens in their host plants [38,39]. Research indicates that DSF-mediated communication regulates a wide array of genes, including those involved in biofilm formation and the production of extracellular polysaccharides, thereby enhancing the bacterium’s ability to colonise and infect host plants [40,41].

Still, DSFs are also produced by other bacteria of agricultural interest [42] such as *Xanthomonas oryzae* pv. *oryzae* [43], *X. axonopodis* pv. *glycines* [44], *X. citri* subsp. *citri* [45], *Burkholderia cepacia* [46], *B. glumae* [47], and *B. plantarii* [48]. (Table 1), and by bacteria of non-agricultural interest, such as *Pseudomonas aeruginosa*, *Cronobacter turicensis*, *Leptospirillum ferrooxidans*, *Leptospirillum ferriphilum*, *Lysobacter enzymogenes*, *Stenotrophomonas maltophilia,* and *Leptospirillum brunescens* [49].

The functions of DSFs can vary significantly depending on the specific bacterial species involved. In *X. oryzae* pv. *oryzae*, the DSFs play a pivotal role in promoting bacterial attachment to surfaces and facilitating biofilm formation. Rai et al. [50] demonstrated that DSFs enhance the ability of *X. oryzae* to adhere to plant surfaces, which is a critical step in establishing infection. The biofilm formed as a result of DSF signalling provides a protective environment for the bacteria, allowing them to survive in adverse conditions and maintain virulence [2]. Similarly, in *X. axonopodis* pv. *glycines*, DSFs have been shown to regulate biofilm formation. Thowthampitak et al. [44] reported that DSFs promote biofilm development and suppress the production of type II effectors, including cellulase, lipase, xylanase, and cellobioseidase. This suppression of effector production is significant as it may alter the bacterium’s pathogenicity, potentially allowing it to evade host defences while establishing a successful infection [1,3]. In *X. citri* subsp. *citri*, the response to DSFs is more nuanced. Beaulieu et al. [37] and Caserta et al. [51] found that gene expression in this species is exceptionally responsive to specific DSFs, such as 11-methyldecenoic acid and cis-2-dodecenoic; however, it was noted that the bacterium is insensitive to other signal molecules, such as 2-tetradecenoic acid, which are characteristic of *Xf*. This specificity in response suggests that different DSFs can elicit distinct regulatory pathways, influencing the overall behaviour and pathogenicity of *X. citri* [4,52]. The genus *Burkholderia*, including species such as *B. cepacia*, *B. glumae*, and *B. plantarii*, uses DSFs to regulate biofilm formation positively. Horgan et al. [53] highlighted that DSFs in these species enhance biofilm development, which is critical for their survival and pathogenicity. The synthesis of DSFs in *Burkholderia* species is dependent on the RpfF protein, as noted by Ryan et al. [25], indicating a conserved mechanism of DSF production across different bacterial genera (Table 2).

### 2.2. DSF: Regulation of Biofilm and Bacterial Virulence

The regulation of biofilm formation and virulence in phytopathogenic bacteria is significantly influenced by DSFs, which are key components of QS systems. DSFs are related to a cluster of proteins known as the regulation of pathogenicity factors (Rpf), which includes RpfF (the enzyme responsible for DSF synthesis), RpfC (the membrane sensor that detects DSFs), and RpfG (the response regulator activated by RpfC) [54,55]. The synthesis of DSFs occurs through the enzymatic action of RpfF, which catalyses the dehydration of 3-hydroxyacyl fatty acid (ACP) intermediates, releasing DSFs into the environment [56]. The concentration of DSFs is directly correlated with bacterial population density, leading to a robust signalling mechanism that allows bacteria to coordinate their behaviour in response to environmental cues [57]. Once released, DSFs diffuse into the surrounding environment and are detected by neighbouring bacterial cells through specific receptors, primarily RpfC.

**Figure 2:**
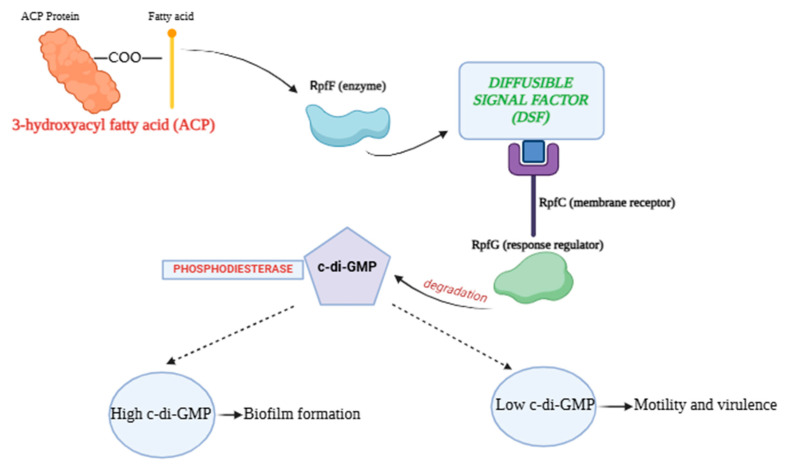
Schematic representation of diffusible signal factors (DSFs) synthesis and activity.

Upon binding to DSFs, RpfC undergoes phosphorylation, which activates RpfG, leading to the degradation of cyclic di-GMP (c-di-GMP) [58]. This degradation is crucial because c-di-GMP levels play a pivotal role in regulating various bacterial functions, including biofilm formation and the expression of virulence factors. Low levels of c-di-GMP promote motility and the expression of virulence genes, while high levels typically enhance biofilm formation and the production of EPS [59,60] (Figure 2). Thus, DSFs act as signalling molecules that modulate the balance between motility and biofilm formation, influencing the overall pathogenicity of the bacteria [46].

In specific pathogens, the role of DSFs in biofilm regulation varies. Some Gram-negative bacteria have the ability to form biofilms after a previous planktonic phase and do so to protect themselves from abiotic and biotic stresses. For instance, in *X. oryzae* pv. *oryzae*, DSFs enhance bacterial attachment and biofilm formation [61]. Conversely, in *X. campestris*, DSFs negatively regulate biofilm formation by suppressing the expression of genes involved in polysaccharide synthesis, thereby inhibiting the initial aggregation necessary for biofilm development [62]. This dual role highlights the complexity of DSF signalling and its context-dependent effects on bacterial behaviour. In this context, the concept central to Tao et al.’s research [51] revolves around the use of DSF signals in the transition between biofilm and planktonic growth. Their findings demonstrated that DSF signals play a crucial role in regulating this transition, particularly in the absence of the ManA protein. The introduction of DSF significantly reduced biofilm growth, while biomass remained constant throughout bacterial growth. Furthermore, when DSF was added after six hours of development, biofilm biomass accumulated more slowly, reaching a plateau after three hours. These results further support the complex regulatory role of DSF in bacterial biofilm dynamics and appear closely tied to other important aspects of bacterial behaviour, such as the regulation of virulence factors. Other studies have shown that DSFs can influence the expression of various virulence-associated traits, including the production of extracellular enzymes and toxins [63]. For example, in *B.cepacia*, DSFs regulate the expression of virulence factors through similar mechanisms involving c-di-GMP turnover [64]. This suggests that DSFs are integral to the pathogenicity of multiple bacterial species, facilitating their adaptation to host environments and enhancing their survival and infectivity (Table 2).

**Table 2:** Role and mechanisms of action of diffusible signal factors (DSFs) in phytopathogenic bacteria.

Bacteria	Role of DSFs	Specific Mechanism	Other Info	Ref.
*Xanthomonas campestris* pv. *campestris*	Negatively regulates biofilm production	RpfC/RpfG system	- xagABC gene involved in encoding glycotransferases; - DSF synthesis depends on RpfF; - RpfF/RpfC interact directly; - *Xcc*Rpf/*Xcc*DSF: DSF accumulation and protease expression in the stationary phase; - DSF signal regulated by RpfC; - Hpt domain of RpfC is important for regulation; - RpfH gene present and functional for modulation;	[16,25,31,45,65,66,67,68,69,70,71]
*Xylella fastidiosa*	Promotes bacterial adhesion to surfaces and promotes biofilm production and regulates virulence factors	RpfC/RpfG system	- RpfF is involved in the production and detection of DSF; - RpfF inhibits RpfC and vice versa; - DSF-regulated adhesin is expressed early in the bacterial growth initiation phase - The Hpt domain has a different structure to that of *Xcc*; - The RpfH gene is absent;	[1,37,66,72,73,74,75,76,77,78,79]
*Xanthomonas oryzae* pv. *oryzae*	- Promotes bacterial attachment to surfaces and biofilm formation; - suppresses the motility of the bacterium and the production of type II effectors: cellulase, lipase, xylanase and cellobioseidase	RpfC/RpfG system	DSF synthesis depends on RpfF	[50,80]
*Xanthomonas axonopodis* pv. *glycines*	Regulates biofilm formation and virulence factors	RpfC/RpfG system	DSF synthesis depends on RpfF	[44]
*Xanthomonas citri* subsp. *citri*	They have the same functions as in *Xcc*	RpfC/RpfG system	- Gene expression more responsive to molecules such as 11-methyldecenoic acid and insensitive to molecules such as 2-tetradecenoic acid characterising *Xf*; - DSF synthesis depends on RpfF	[37,51]
*Burkholderia cepacia*, *Burkholderia glumae*, *Burkholderia plantarii*	Promotes bacterial adhesion to surfaces and promotes biofilm production	RpfC/RpfG system	DSF synthesis depends on RpfF	[25,53]

## 3. Role of DSF in *Xf*

### 3.1. Virulence Mechanisms and Regulation of the Quorum Sensing System

*Xf* virulence is strongly linked to its ability to colonise xylem vessels, where it forms a biofilm that hinders sap flow [81], resulting in severe water stress and nutritional deficiency in host plants. This colonisation process is facilitated by the production of DSFs, specifically cis-2-tetradecenoic acid and cis-2-hexadecanoic acid, which are necessary for the regulation of virulence factors through an Rpf protein system-mediated QS mechanism [31,37].

These signals enable *Xf* to control its behaviour, namely in biofilm formation and virulence factor expression, in response to changes in cell density [75,82]. The study by Chatterjee et al. [75] highlights that the signal transduction pathway of DSFs can be highly divergent between bacterial taxa, even though the same Rpf proteins are involved in DSF sensing. For instance, in *Xcc*, RpfF and RpfC interact directly, whereas, in *Xf*, this interaction appears to be bidirectional, with RpfF inhibiting RpfC and vice versa [83] (Table 2). The fact that this variation suggests the regulatory mechanisms modulation of DSF signalling in these two bacteria are not identical is probably due to adaptation into different ecological niches and host interactions. DSF accumulation in *Xf* leads to the expression of adhesin proteins that facilitate the attachment of the bacteria to surfaces, a requisite step for biofilm. Unlike what occurs in *Xcc*, where both the accumulation of DSF and protease production happen in the early stationary growth stage, *Xf* is more rapid in its response to the exogenous addition of DSF addition and results in early production of DSF-regulated adhesins [37,75]. This fast response indicates *Xf*’s adaptation mechanisms, which allow it to infect its host plants more effectively. Moreover, the production and regulation of DSF signals in *Xf* are influenced by various factors, including the type of culture medium and environmental conditions. The RpfF enzyme is able to utilise other substrates to produce various kinds of DSFs, which further complicates the regulation of the landscape of DSF signalling [82,84]. In *Xcc*, RpfC regulates DSF production by inhibiting it at low cell densities, a role that is non-functional in *Xf*, showing that *Xf* has evolved a different control system for maximizing its pathogenic potential [75,82]. The pathogenicity of *Xf* is also accentuated by their role in enhancing the development of biofilms, which is critical for the bacterium’s survival and pathogenicity. Biofilms establish a protective micro-niche for bacterial cells that raises their levels of resistance against environmental stresses as well as against host defences [38,85]. *Xf*’s ability to form biofilms directly correlates with its production of DSF because the signalling compounds induce bacterial cell clumping as well as a permanent biofilm structure [27,86]. Interaction with biofilm establishment and DSF signalling is the key to success for *Xf* as a phytopathogen in terms of successfully colonising xylem vessels and evading the host’s immune system. Overall, the pathogenesis mechanisms of *Xf* are based mainly on its ability to synthesise and function in response to DSFs using a complex system of QS. The DSF signalling and regulation differences between *Xf* and other closely related bacteria, such as *Xcc*, reflect the evolutionary processes that allow *Xf* to live and survive in its specific ecological niche.

### 3.2. Role of the Rpf System in Xf

In *Xf*, RpfF produces different amounts of DSF, and some RpfF mutants lacking DSF have tremendous trouble infecting insects and forming biofilms. C-di-GMP is also involved in biofilm formation and the regulation of virulence determinants, with the difference that in *Xf,* it appears to inhibit virulence induction. In contrast, in *Xcc* it induces biofilm dispersal (*yybA*, *rpoH*, *glnB*, *colR*, *rpfE*, *rpoD*) and sigma factors for regulating expression according to environmental conditions [61,87]. In addition to this, RpfF also regulates the expression of adhesin genes and also genes that code for haemagglutinins, i.e., hxfA and hxfB; so it happens that in bacteria where RpfF is inactive, the expression of genes decreases and hence the bacterium cannot adhere and form biofilms, but they have more developed pili, which make them more motile. This suggests that Xf, without RpfF, displays greater mobility when exploring the plant and is less effective at forming biofilms [61,71]. *Xf* can produce various types of DSF molecules, implying that the roles of RpfF depend on the environment in which the bacterium proliferates [27]. This indicates that the system responsible for DSF production adapts to the environment to streamline the bacterium’s behaviours, thereby facilitating appropriate responses to stimuli from its particular habitat [1]. Besides RpfF, another component is added to the molecular mechanism, i.e., RpfC, which interacts with RpfF and is responsible for regulating the expression of the genes responsible for exopolysaccharide production and detecting the presence of DSF [76,79]. RpfC’s histidine phosphotransfer (Hpt) domain play a role in regulation but in *Xf* is structurally different from that in *Xcc*, indicating adaptation to a more specialized function. In *Xcc*, Hpt acts primarily with RpfG to regulate EPS and enzyme production [81]. In *Xf*, unlike *Xcc*, there is no rpfH gene, where its functional operation is controlled by other factors such as RpfF (for DSF production), RpfC and RpfG (for transduction and regulation) [72,73,74,78].

The Rpf system also colonises the bacterium in the vector insect, while the RpfF mutant cannot colonise insects. Therefore, the bacterium cannot stabilise, there is no biofilm formation in the vector, and transmission does not occur [72]. The RpfC mutant can form the biofilm but is ineffective in transmitting the bacterium. This suggests that although the cell–cell signalling mechanism and DSFs regulating virulence and biofilm formation are the same in Gram-negative bacteria, certain functions differ from one another to fulfil distinct requirements.

## 4. Application of DSFs for *Xf* Containment

*Xf* is a significant pest risk all over the world because it results in tremendous economic and environmental damage. It is extremely hard to manage despite the agronomic, mechanical, chemical and biological control techniques that have been created over the years [88]. Novel and environmentally friendly control methods include the use of DSFs to mitigate the effects of *Xf* without employing invasive measures, such as quorum quenching (QQ) [89] and “pathogen confusion” [90].

### 4.1. Quorum-Quenching Strategy

Quorum-quenching (QQ) strategy refers to a novel approach founded upon interference with bacterial communication systems via enzymatic (QQ enzymes) or chemical (QSI inhibitors) means [91]. Enzymes are able to degrade or inactive signal molecules AHL released by bacteria to inhibit the formation of biofilms or virulence production [92]. They belong to three general classes: lactonases [93], amylases [94] and oxidoreductases [95].

Lactonases possess various specificities since they deactivate short- and long-chain AHL by hydrolysing the ester bond of the lactone ring to yield acyl-homoserine [93]. Nevertheless, certain lactone substrates like δ-, ε-, and γ-lactones are active only at high temperatures [96,97,98].

There exist four kinds of lactonases:

1. The metallo-β-lactamase-like lactonases: found in bacteria, archaea and eukaryotes, their principal trait is metal binding that renders them effective with a substrate preference for AHL [73,96,97,99];
2. The phosphotriesterase-like lactonases found in bacteria and archaea are thermostable metals with a preference for AHL with long acyl chains [100];
3. The α/β hydrolase fold lactonases found in bacteria display α/β hydrolase folding [101];
4. The paraoxonases: found in bacteria, display a β-helix fold [102] and hydrolyse a range of substrates, including AHLs with lengthy acyl chains [103].

On the other hand, acylases are classified under the family N-terminal nucleophilic hydrolases. Their αβ/βα structural organisation distinguishes them. Their three-dimensional structure indicates that they prefer long-chain acyl AHLs [104,105] by hydrolysing their amide bond of these compounds [106].

Conversely, the oxidoreductases do not change the concentration of the signal molecule; instead, they modify it through the oxidation of the carbon atom in the acyl chain of the AHLs or even by reducing the carboxyl group to a hydroxyl group, so they are able to control the QS response [100].

The enzymes mentioned earlier do not modify the development of microorganisms [107,108], and they are stronger than QSIs, which also have different cytotoxicity [109,110].

In order to be effective, a QSI must fulfil specific requirements [111,112,113]. In particular, it should be a small molecule that can effectively reduce QS-regulated gene expression, exhibit high specificity for a given regulator without causing adverse effects on bacteria or host, be chemically stable and resistant to metabolic degradation, as well as possess a preferably longer length than native AHL. Due to these properties, bacteria are unlikely to develop resistance to the treatment, as these compounds do not exert significant selective pressure and do not alter the host’s population of beneficial bacteria [114]. QSIs include plant extracts as they have structural similarity to QS signals (AHL) and are capable of degrading signal receptors [113,115]. Examples of plant extracts are *Combretum albiflorum*, *Laurus nobilis,* and *Sonchus oleraceus,* which have shown anti-QS activity [116]. However, their production occurs in low concentrations and, in some cases, may be associated with toxicity, thus representing a significant limitation. One possible way to overcome these difficulties is the chemical synthesis of these signals to produce synthetic analogues [117].

Beyond these key features, the QQ strategy has both advantages and disadvantages (Table 3): it is still uncommon although effective because it replaces the use of antibiotics in both agricultural and other fields [91,92] by inhibiting bacterial virulence through lighter selective pressure to avoid killing bacteria [92,118,119,120]. However, it could affect bacteria that positively affect the plant [91,92]. It is not a strategy towards which bacteria develop resistance except for so-called “social cheaters” as they interfere with QQ by interrupting the formation of QS signals [121]. Resistance is a characteristic that depends on various aspects, such as the type of strategy used and the type of inhibitor [121] and also distinguishes enzymes where, in this case, it is given by a high production of QS autoinducers that act against hydrolysis by QQ enzymes [92,108,120,122].

Research on DSF-dependent bacteria in the context of QQ studies is scarce probably because the DSF biosensor, compared to the AHL biosensor, is less reliable and less sensitive, thus limiting the identification of enzymes capable of modifying or degrading DSF. The enzymes currently known are carbamoyl phosphate synthase (identified in *Pseudomonas aeruginosa*, of non-agricultural interest) [123] and RpfB, which plays an important role in DSF turnover in *Xcc* and *X*. *oryzae* pv. *oryzae* [124] because it converts DSF to fatty acyl-CoA, which is then degraded by oxidation. In this way, RpfB influences QS processes and regulates certain behaviours, such as virulence [125].

### 4.2. Pathogen Confusion

Genetically transforming plants to contrast *Xf* may be another option to the QQ method, but not much is understood about which. Earlier experiments have proved the presence of an RpfF mutant of *Xf* that is incapable of spreading from plant to plant and incapable of transmission by vectors as they are incapable of forming biofilm in the insect. The virulence of the mutants is linked to the operation of the density-dependent signalling system used to reduce the virulence of *Xf* at high cell densities [126]. Application of this technique ‘confounds’ the pathogen by generating surplus DSF in transgenic plants [90].

In the study conducted by Lindow et al. [90] to demonstrate the effects on *Xf* virulence caused by the overexpression of DSF, RpfF was overexpressed at high levels by inserting this gene into the “Freedom” grape variety, which was found to be susceptible to Pierce’s Disease caused by *Xf*. This gene was introduced into the plants through a gene transformation process, and it showed that transgenic plants with the RpfF gene had fewer diseased leaves than non-transformed plants. Several weeks later, the original “Freedom” plant had around eight diseased leaves per plant, while the transgenic plants had about two during the initial growth phase. To study this in detail, a specific transgenic line called “FT2” was employed, which showed that the symptoms spread much more slowly and stopped after about 8–9 weeks, whereas in the non-transformed plant, the symptoms kept worsening. They proceeded to check if the disease reduction in FT2 grape is linked to DSF production and reported that transgenic plants in both the xylem sap and the leaves produce DSF and that this does not occur in the native plant. The resulting DSF molecules produced are numerous and are capable of inhibiting the potential bacterium *Xf*’s communication or virulence. Caserta et al. [51] also conducted research on this topic using the RpfF gene of *Xf* (strain 9a5c) that was amplified and cloned in *Escherichia coli*, then used for directional cloning in *Agrobacterium tumefaciens*, in order to transform *Citrus sinensis* × *Poncirus trifoliata* plants. The transformed plants were verified by PCR and qPCR, confirming the expression of the RpfF gene. The analyses showed that the transformed plants had smaller lesions and less proliferation of the pathogen *X. citri* subsp. *citri*, suggesting a reduction in susceptibility due to the alteration of RpfF-mediated DSF signalling. Furthermore, they also showed a reduced expression of *X. citri* subsp. *citri* virulence genes, with lower bacterial motility and smaller microcolonies. Mechanical inoculation further limited virulence, probably due to the jasmonic acid-mediated defence response. Thus, in conclusion, RpfF gene expression reduced the severity of infection, suggesting potential applications in disease control [51].

## 5. Future Prospects: Challenges and Limitations of Quorum Quenching and Transgenic Plant Strategies

The management of *Xf*, a key plant pathogen affecting numerous crops, has been at the centre stage since it poses the risk of destabilizing plant productivity. New strategies, such as disrupting QS and generating transgenic plants that excrete more DSFs, have emerged as potential means of disease control. These alternatives, however, have diverse limitations and complications that must be addressed before they can be reliable methods to employ in agricultural production.

QQ strategies utilize enzymes capable of breaking down signalling molecules to disrupt bacterial communication and virulence. Experimental studies have proven the potential for QQ enzymes, although. One of the key challenges is the stability of the enzymes; QQ enzymes are likely to degrade rapidly under field conditions due to environmental factors such as temperature changes, pH changes, and exposure to UV light [89,127]. To enhance their lifespan as well as their efficiency, more stable forms of enzymes or encapsulation techniques must be synthesized through research [89].

Also, the specificity of QQ enzymes is extremely crucial. Degradation of DSF signals can indirectly affect beneficial bacteria that rely on homologous signalling networks, potentially disrupting the microbial community of plants [128,129]. Future research should be on developing highly discriminatory enzymes against *Xf* DSFs and not against beneficial microorganisms [130]. In addition, delivery systems for QQ enzymes are problematic; effective methods can be foliar sprays, soil applications, or microbial consortia that naturally produce QQ enzymes [131,132]. Identifying the most effective and sustainable delivery systems will be pivotal to successfully deploying QQ strategies in agricultural settings.

Plant genetic modification to have high levels of DSFs aims to “deceive” *Xf* and reduce its virulence. While tests in the lab and greenhouse proved the efficacy of this action, there are nevertheless some issues of concern. One of the main concerns relates to the long-term ecological impact of extended DSF production in transgenic plants, which might have unforeseen consequences such as alterations in plant physiology or effects on other microbes that are associated with the plant [3,133]. Systematic studies have to be conducted to ascertain potential trade-offs between *Xf* resistance and other agronomic traits [27].

Another concern is the capacity of *Xf* to adapt. The bacterium is highly versatile, and repeated exposure to high concentrations of DSF can cause the emergence of resistant strains that are not susceptible to QS interference [134,135]. Monitoring the adaptation of *Xf* populations to DSF-based treatments will be critical to ensure the long-term efficacy of this approach [136]. Furthermore, regulatory and public acceptance concerns of genetically modified (GM) crops remain high, particularly in nations like Europe, where GM crops are heavily regulated [137]. Having alternative, non-GM approaches that use natural or induced DSF overproduction in plants may be a more palatable solution [27,128]. Excessive production of DSF in crops is feasible through non-GM technology, such as non-transgenic genome editing. Such techniques aim to modify the plant genome without foreign DNA, hence making it more acceptable to the public and less regulated. One example of such a tool is preassembled CRISPR/Cas9 ribonucleoproteins that can be delivered into plant cells to induce specific genetic alterations without the need to introduce a foreign gene. This has been noted in a review of non-transgenic genome editing techniques in crops [138]. An example is when lipofection transfects plant cells with the CRISPR/Cas9 protein complex. Liposomes, in this process, deliver the protein complex into the cell without including foreign DNA, thereby avoiding the production of genetically modified organisms (GMOs). It has been tailored to enhance crops without adding foreign DNA, as described by Zhang et al. [139].

These methods are promising ways of achieving DSF overproduction in plants through non-transgenic genome editing techniques, offering more universally accepted solutions to crop improvement. While advances have been made in elucidating DSF-mediated signalling and *Xf* pathogenesis, numerous basic questions remain unanswered. Among these is how the DSFs operate within the plant microbiome as a whole. Understanding how DSFs function in microbial communities may unveil the key to using beneficial bacteria to enhance natural QQ systems [95]. Identification of new QS inhibitors aside from enzymatic degradation could also expand the arsenal of *Xf* management [127,140].

Field-scale validation of DSF-based strategies is another future research priority, as most research has been conducted in controlled environments. Large-scale experiments are needed to validate the real-world applicability and effectiveness of these strategies [131,141]. Synthesizing DSF-targeting with other disease control practices—i.e., vector management, resistant crop varieties, and sustainable agronomic practices—will be called upon to control the disastrous effects of *Xf* on global agriculture [136,142].

Finally, both QQ and transgenic plant strategies offer promising alternatives for controlling plant pathogens and reducing chemical pesticide dependence. However, each approach presents specific limitations. QQ strategies may affect beneficial microbes, be influenced by environmental factors, and face challenges from bacterial “social cheaters”. Transgenic plants, on the other hand, risk the evolution of pest resistance, gene transfer to wild relatives, and potential ecological concerns. Table 4 summarizes the key advantages and challenges of both strategies.

## 6. Conclusions

DSFs are the hub of *Xf*’s quorum sensing system, and they regulate important functions like biofilm formation, motility, and the expression of virulence factors. Unlike all other bacterial QS systems, *Xf*’s DSF-based signalling has a twofold role: it promotes adhesion in the xylem while promoting dispersal through insect vectors. Hence, it plays a critical role in the epidemiology of the bacterium. An understanding of how DSFs function to govern such activities will be crucial to designing targeted control strategies.

Two of the most promising approaches are QQ, which blocks and disrupts DSF signalling to suppress bacterial virulence and the genetic engineering of transgenic plants that overexpress DSFs to modify *Xf* behaviour. These new strategies offer alternatives to conventional control methods, providing more sustainable and environmentally friendly options.

More research will have to study the molecular interaction between DSFs and host plants, identify novel DSF-degrading enzymes, and optimise biotechnological approaches for interference with QS pathways. Advancing our knowledge could lead us to develop improved, sustained means to lower *Xf* infection and enhance the agricultural resistance against this devastating pathogen.

## Figures and Tables

**Table 1:** DSFs identified in some phytopathogenic bacteria.

Bacteria	Main Host	DSFs	Ref.
*Xanthomonas campestris* pv. *campestris*	Crucifers	Cis-11-methyldodecenoic acid; Cis-2-dodecenoic acid; Cis, cis-11-methyldodeca-2,5-dienoic acid; Cis-10-methyl-2-dodecenoic acid; Cis-9-methyl-2-dodecenoic acid; Cis-2-undecenoic acid	[31]
*Xanthomonas oryzae* pv. *oryzae*	Rice	Cis-11-methyl-dodecenoic acid; Cis-2 dodecenoic acid; Cis, cis-11-methyldodeca-2,5-dienoic acid	[43]
*Xanthomonas axonopodis* pv. *glycines*	Soybean	Cis-11-methyldodecenoic acid	[44]
*Xanthomonas citri* subsp. *citri*	Citrus	Cis-2-dodecenoic acid	[45]
*Burkholderia cepacia*	Onion	Cis-2-dodecenoic acid	[46]
*Burkholderia glumae*	Rice	Cis-2-dodecenoic acid	[47]
*Burkholderia plantarii*	Rice	Cis-2-dodecenoic acid	[48]
*Xylella fastidiosa*	Grapevine, citrus, almond, olive etc.	2-tetradecenoic acid 2-cishexadecanoic acid	[31]

**Table 3:** Advantages and disadvantages of Quorum Quenching (QQ).

QQ Advantages	QQ Disadvantages
Replaces the use of antibiotics [91,92];	It affects the bacteria that have a positive effect on the plant [91,92];
The light selective pressure applied serves to inhibit bacterial virulence without killing microorganisms [92,118,119,120];	The type of inhibitor and the type of strategy used affect resistance to QQ [121];
Bacteria do not develop resistance to QQ strategies except for “social cheaters” [121];	Bacteria “social cheaters” negatively interfere with QQ strategies because they interrupt the formation of QS signals [121];
Enzymes less inclined to resistance [92,107,108,120,122].	High production of QS autoinducers causes enzyme resistance [92,108,120,122].

**Table 4:** Advantages, challenges, and potential solutions of the Quorum Quenching (QQ) and transgenic plant strategies.

Aspect	Quorum Quenching (QQ) Strategies	Transgenic Plant Strategies
**Objective**	Interfere with bacterial communication (QS) and reduce the virulence of *Xf*.	Genetically modify plants to produce DSF and reduce the virulence of *Xf*.
**Mechanism**	Enzymes (lactonases, acylases) are used to degrade QS signals and disrupt bacterial virulence.	Genetically engineered plants to produce high levels of DSF, “deceiving” *Xf* and reducing its virulence.
**Key Challenges**	- Enzyme stability: QQ enzymes degrade quickly in the field due to environmental factors (temperature, pH, UV). - Specificity: Enzymes must be specific to *Xf* to avoid harming beneficial bacteria. - Delivery systems: Effective distribution methods (foliar sprays, soil applications, microbial consortia) need to be developed.	- Long-term ecological impact: Extended DSF production may affect plant physiology or beneficial microbes. - Resistance development: *Xf* could develop resistance to DSF production. - Public acceptance: GM crops are highly regulated and face opposition, especially in Europe.
**Proposed Solutions**	- Develop more stable QQ enzymes through research and encapsulation techniques. - Create highly specific enzymes for *Xf*. - Identify the most effective delivery systems for QQ enzymes in agricultural settings.	- Systematic studies on the ecological impacts of DSF production in transgenic plants. - Monitor long-term *Xf* resistance. - Research alternative methods and non-GM approaches (e.g., genome editing).
**Alternative Techniques**	- Genome editing (e.g., CRISPR/Cas9) can be used to induce DSF overproduction in plants without introducing foreign DNA, reducing public opposition.	- Genome editing techniques like preassembled CRISPR/Cas9 ribonucleoproteins can induce specific genetic changes in plants without creating GMOs.
**Future Research Directions**	- Understand how DSFs function in plant microbial communities to improve natural QQ systems. - Identify new QS inhibitors to expand *Xf* management tools. - Field-scale validation of DSF-based strategies.	- Validate the efficacy of transgenic plants in real-world and field environments. - Combine DSF-based strategies with other disease control practices (vector management, resistant crop varieties, sustainable agronomy).

## Data Availability

No new data were created or analyzed in this study. Data sharing is not applicable to this article.

## Abbreviations

*Xf*	*Xylella fastidiosa*
DSF	Diffusible signal factor
QS	Quorum sensing
QQ	Quorum Quenching
AHL	Acyl-homoserine lactone
*Xcc*	*Xanthomonas campestris* pv. *campestris*
EPS	Extracellular polysaccharide
ACP	3-hydroxyacil fatty acid
c-di-GMP	Cyclic di-GMP
Hpt	Histidine phosphotransfer
GM	Genetically modified
GMO	Genetically modified organism
